# Accounting for symptom heterogeneity can improve neuroimaging models of antidepressant response after electroconvulsive therapy

**DOI:** 10.1002/hbm.25620

**Published:** 2021-08-13

**Authors:** Benjamin S. C. Wade, Gerhard Hellemann, Randall T. Espinoza, Roger P. Woods, Shantanu H. Joshi, Ronny Redlich, Udo Dannlowski, Anders Jorgensen, Christopher C. Abbott, Leif Oltedal, Katherine L. Narr

**Affiliations:** ^1^ Ahmanson‐Lovelace Brain Mapping Center, Department of Neurology UCLA Los Angeles California USA; ^2^ Department of Psychiatry and Biobehavioral Sciences, David Geffen School of Medicine UCLA Los Angeles California USA; ^3^ Institute of Translational Psychiatry, Department of Mental Health University of Münster Münster Germany; ^4^ Department of Clinical Psychology University of Halle Halle Germany; ^5^ Psychiatric Center Copenhagen Copenhagen Denmark; ^6^ Department of Psychiatry University of New Mexico School of Medicine Albuquerque New Mexico USA; ^7^ Department of Clinical Medicine University of Bergen Bergen Norway; ^8^ Mohn Medical Imaging and Visualization Centre, Department of Radiology Haukeland University Hospital Bergen Norway

**Keywords:** electroconvulsive therapy, machine learning, major depressive disorder, structural neuroimaging, symptom heterogeneity

## Abstract

Depression symptom heterogeneity limits the identifiability of treatment‐response biomarkers. Whether improvement along dimensions of depressive symptoms relates to separable neural networks remains poorly understood. We build on work describing three latent symptom dimensions within the 17‐item Hamilton Depression Rating Scale (HDRS) and use data‐driven methods to relate multivariate patterns of patient clinical, demographic, and brain structural changes over electroconvulsive therapy (ECT) to dimensional changes in depressive symptoms. We included 110 ECT patients from Global ECT‐MRI Research Collaboration (GEMRIC) sites who underwent structural MRI and HDRS assessments before and after treatment. Cross validated random forest regression models predicted change along symptom dimensions. HDRS symptoms clustered into dimensions of somatic disturbances (SoD), core mood and anhedonia (CMA), and insomnia. The coefficient of determination between predicted and actual changes were 22%, 39%, and 39% (all *p* < .01) for SoD, CMA, and insomnia, respectively. CMA and insomnia change were predicted more accurately than HDRS‐6 and HDRS‐17 changes (*p* < .05). Pretreatment symptoms, body‐mass index, and age were important predictors. Important imaging predictors included the right transverse temporal gyrus and left frontal pole for the SoD dimension; right transverse temporal gyrus and right rostral middle frontal gyrus for the CMA dimension; and right superior parietal lobule and left accumbens for the insomnia dimension. Our findings support that recovery along depressive symptom dimensions is predicted more accurately than HDRS total scores and are related to unique and overlapping patterns of clinical and demographic data and volumetric changes in brain regions related to depression and near ECT electrodes.

## Graphical abstract

Depression symptom heterogeneity limits the identifiability of treatment‐response biomarkers. We developed machine learning models to predict symptom change along latent dimensions of depression following treatment with electroconvulsive therapy in a large, multisite cohort using a combination of neuroimaging, clinical, and demographic predictors. Recovery along homogenous latent symptom dimensions was predicted more accurately than change along standard, more heterogeneous total score measures.

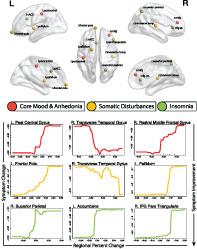

## 1 INTRODUCTION

Depression is a leading cause of disability worldwide (James et al., 2018). Despite its prevalence, effective treatment remains challenging. Antidepressant and behavioral interventions serve as first‐line treatments but roughly one third of patients remain unresponsive to these interventions (Trivedi et al., 2006). Heterogeneity of depressive symptoms likely contributes to treatment failures as a variety of mechanisms and etiologies may be associated with particular dimensions of depressive symptoms.

Limited alignment between DSM‐based categorizations of psychiatric disorders and underlying neurobiological processes related to these disturbances has motivated an effort to characterize these dysfunctions along symptom dimensions. The National Institutes of Mental Health's Research Domain Criteria (RDoCs) has attempted to formalize this research. In support of this approach, a high degree of symptomatic heterogeneity is often observed across individuals sharing the same diagnosis. A DSM‐V depression diagnosis, for example, requires presentation of at least five out of nine symptoms in addition to one of two core symptoms; yielding 227 potential symptom constellations for a single diagnosis (van Loo, de Jonge, Romeijn, Kessler, & Schoevers, 2012). Moreover, there may be nuanced differences in neural systems involved across this potentially varied spectrum of symptom manifestations. Thus, a clearer mapping between neural systems and symptom constellations would inform more targeted antidepressant neurostimulation techniques such as transcranial magnetic stimulation, high‐definition transcranial direct current stimulation, or more focal electroconvulsive therapy (ECT) strategies.

ECT is a rapidly acting and effective treatment for major depressive disorder (MDD) boasting response rates between 50 and 80% (Haq, Sitzmann, Goldman, Maixner, & Mickey, 2015; Tokutsu et al., 2013). Previous studies have investigated neuroimaging‐based biomarkers for various clinical outcomes following ECT (Jiang et al., 2018; Leaver et al., 2018; Schmitgen et al., 2020). Among these, Jiang et al. identified separable treatment‐predictive and treatment‐responsive neuroimaging biomarkers of ECT response with gray matter morphometry of the right hippocampus, right orbitofrontal gyrus, left post central gyrus, and left lingual area among treatment predictive markers. Treatment‐related gray matter density increases included the left supplementary motor cortex, left postcentral gyrus, and left precuneus (Jiang et al., 2018). A recent report by Schmitgen et al. identified pre‐ and post‐ECT associations between the cortical thickness of the left rostral anterior cingulate, left medial orbitofrontal cortex, and gyrification of the right middle frontal gyrus, and treatment‐related reductions in depressive symptoms; pretreatment symptom severity was also a key predictor of symptom change (Schmitgen et al., 2020). Previous work from our group reported on volumetric increases in the accumbens, pallidum, and caudate among ECT‐responsive patients (Wade et al., 2016). Using resting state functional connectivity measures, Leaver et al. reported that pretreatment connectivity of networks encompassing the dorsolateral prefrontal cortex, subgenual anterior cingulate, and motor cortex were predictive of ECT response (Leaver et al., 2018).

Many earlier studies tracked the course of depressive symptoms using only aggregate scores from multi‐item scales (Ousdal et al., 2020) such as the Hamilton Depression Rating Scale (HDRS), thereby ignoring potentially separable symptom dimensions. Validity of the HDRS total score and related scales as measures of depression severity have been established, but aggregated scales may fail to separate distinct symptom dimensions (Michael Bagby, Ryder, Deborah Schuller, & Marshall, 2004). Moreover, use of a total score as a primary outcome ignores potentially subtle differences between symptom profiles that might relate to clinical outcomes.

Several strategies could be used to model symptom heterogeneity in the context of biomarker‐identification studies including relating neuroimaging measures to changes in: (1) individual items of a scale; (2) scale sub‐scores related to depression subtypes (i.e., atypical, melancholic, psychotic depression); or (3) weighted combinations of scale items. The first approach is limited as it ignores between‐item correlation and overlooks more parsimonious models. The second approach overcomes this, but, a priori depression subtypes based on clinical observations may not align with clustering tendencies of observed data. The third approach is commonly implemented using either exploratory factor analysis (EFA) or principal components analysis (PCA) and addresses potential limitations of the first two strategies by identifying compact, lower‐dimensional representations of outcomes in a data‐driven manner.

Previous studies used PCA decompositions of the HDRS; however, results have yielded between two to eight factors with inconsistent loadings (Michael Bagby et al., 2004). Others have applied Rasch logistic analysis to extract a subset of six HDRS items (i.e., the HDRS‐6) that provide a consistent unidimensional measure (Bech et al., 1981). Factor analysis and item response theory have also been explored (Faries et al., 2000; Williams, 2001). While these approaches improve psychometric properties of the HDRS by enforcing a more valid unidimensional internal structure, additional information pertinent to other aspects of depression is then discarded by item exclusion.

More recently, we used EFA to identify latent symptom dimensions in a large, multisite patient cohort undergoing electroconvulsive therapy (ECT) for depression (Wade et al., 2020). We identified three symptom dimensions from pretreatment HDRS‐17 items: somatic symptoms, core mood and anhedonia, and insomnia. Here, we build on this work and aim to relate patient demographic, clinical, and patterns of regional ECT‐induced volumetric brain changes to changes in latent symptom dimensions using data‐driven methods. We hypothesized that demographic and clinical measures would be broadly related to symptom changes, and as has been previously reported, that regional volumetric changes of the hippocampus, motor cortex, striatum, and cingulate would be commonly related to changes along separate symptom dimensions. However, at the same time we hypothesized that there would be unique volumetric changes related to individual symptom dimensions based on the assumption that these dimensions recruit different functional networks. Finally, we hypothesized that prediction of symptom changes would be more accurate along latent symptom dimensions rather than the HDRS‐17 total score. Characterizing these unique and shared predictors and mechanisms of treatment response will inform development of targeted interventions such as neurostimulation techniques capable of targeting specific neural circuits and thus directly targeting important dimensions of depression.

## 2 METHODS

### 2.1 Patients

All 110 patients with depression (*N* = 67 female; age = 52.23 ± 14.71) and 23 unaffected controls (*N* = 15 female; age = 44.34 ± 11.52) were drawn from the Global ECT‐MRI Research Collaboration (GEMRIC) database. Patients were diagnosed with a major depressive episode at the time of study entry and were drawn from four independent sites; control participants were only available from one site. Depressive symptoms for all participants were assessed using the 17‐item Hamilton Depression Rating Scale (HDRS). For patients, the HDRS was recorded approximately 24 hr prior to their first ECT session and again after completing the full ECT index series. Control participant symptoms were recorded twice, approximately 6 weeks apart; approximately the same interval as patients from Site 4. Patients with comorbid disorders included nine with bipolar disorder and 19 with psychotic features. The primary diagnosis of 96 patients was recurrent MDD. Patient clinical, demographic, and treatment information is summarized in Table 1.

**TABLE 1:** Demographic and clinical features

	Patients					Controls	*p* (*T* or $\chi^{2}$)
	Total	Site 1	Site 2	Site 3	Site 4	Site 4	
*N*	110	27	39	16	28	23	
Age, mean (*SD*) years	52.2 (14.7)	40.9 (14.7)	64.4 (9.0)	52.1 (10.8)	46.1 (10.2)	44.4 (11.5)	<.001
Male/female	43/67	14/13	13/26	5/11	11/17	8/15	>.05
BMI, mean (*SD*)	25.9 (5.3)	25.5 (4.4)	26.3 (5.7)	23.6 (4.9)	27.2 (5.9)	24.7 (5.0)	>.05
*Clinical info*							
RUL, primary/secondary	90/2	25/0	37/0	0/2	28/0	—	<.001
BT, primary/secondary	18/19	1/11	2/5	15/0/1 N/A	0/3	—	<.001
LART, primary/secondary	1/0	1/0	0/0	0/0	0/0	—	—
BF, primary/secondary	0/1	0/1	0/0	0/0	0/0	—	—
RUL number, mean (*SD*)	9.2 (5.2)	8.07 (3.6)	10.3 (3.0)	—	13.4 (4.4)		<.001
BT number, mean (*SD*)	6.6 (6.5)	—	7.2 (3.5)	14.2 (4.9)	6.3 (1.5)	—	<.001
ECT device	—	MECTA	Thymatron	Thymatron	Thymatron		—
Titration method	—	Seizure threshold (*n* = 27)	Seizure threshold (*n* = 39)	Seizure threshold (*n* = 2) ½ age (*n* = 14)	½ age (*n* = 28)	—	—
ECT pulse width, milliseconds	—	0.3 (*n* = 25) 0.5 (*n* = 2)	0.25 (*n* = 36) 1 (*n* = 3)	0.5 (*n* = 15) NA (*n* = 1)	0.5 (*n* = 28)	—	—
ECT pulse amplitude, milliamps	—	800 (*n* = 27)	900 (*n* = 39)	900 (*n* = 15) NA (*n* = 1)	900 (*n* = 28)	—	—
Treatment duration, weeks, mean (*SD*)	4.1 (1.4)	3.7 (1.0)	3.7 (1.0)	4.7 (2.1)	4.7 (1.6)	—	—
Antidepressant medication	—	None (*n* = 27)	None (*n* = 2) SSRI (*n* = 17) SNRI (*n* = 16) TCA (*n* = 4)	None (*n* = 3) SSRI (*n* = 4) SNRI (*n* = 3) TCA (*n* = 6)	None (*n* = 5) SSRI (*n* = 4) SNRI (*n* = 15) TCA (*n* = 4)	—	—
Antipsychotic medication, yes/no/NA	—	0/27/0	20/19/0	8/7/1	19/0/9	—	<.001
Benzodiazepines, yes/no/NA	—	0/27/0	16/22/1	8/8/0	0/28/0	—	<.001
Lithium, yes/no/NA	—	0/27/0	0/39/0	1/15/0	4/0/24	—	<.001
Bipolar	9	4	0	5	0	—	<.001
Psychotic features	19	0	16	3	0	—	<.001
Single episode	6	0	2	2	2	—	>.05
Treatment resistant, yes/no, count	104/3	27/0	36/3	13/0/N/A	28/0	—	>.05
HDRS 17 baseline score, mean (*SD*)	24.4 (6.3)	23.8 (6.8)	23.9 (6.9)	28.0 (4.8)	21.7 (4.0)	0.8 (1.7)	<.001
HDRS 17 follow‐up score, mean (*SD*)	11.9 (9.1)	20.0 (9.5)	6.6 (7.1)	13.0 (6.7)	10.9 (6.5)	0.5 (1.5)	<.001

*Note*: Significance tests are applied within the patient group.

Abbreviations: BMI, body mass index; BT, bi‐temporal stimulation; BF, bi‐frontal stimulation; LART, left anterior right temporal stimulation; N/A, not available; RUL, right unilateral stimulation; SNRI, serotonin–norepinephrine reuptake inhibitor; SSRI, selective serotonin reuptake inhibitors; TCA, tricyclic antidepressants.

Treatment protocols were naturalistic as stimulus parameters were not manipulated for research purposes. Local physicians determined each patient's need for ECT. As reported (Wade et al., 2020), several treatment procedures differed across sites. Site 1 used a MECTA Spectrum 5000Q device (Oswego, Oregon); all other sites used Thymatron IV devices (Somatics Inc.). All sites used barbiturate (Methohexitol or Thiopental) induction. Modes of ECT administration were mixed within and across the sites and included right unilateral d'Elia, bitemporal, and left anterior right temporal. Sites 1 and 2 used ultrabrief pulse widths (<0.5 ms) while sites 3 and 4 used brief pulse widths (0.5 ms). ECT pulse amplitude was 800 and 900 milliamps for sites 1 and 2–4, respectively. Only Site 1 tapered patients off antidepressant medications before ECT. Treatment resistance was defined as failure to respond to two or more medication trials at Sites 1 and 2; one or more unsuccessful psychotherapy trials for Site 4; Site 3 had no formalized criteria.

HDRS‐17 assessments and MR imaging occurred immediately before ECT index series and within 2 weeks of completing the index series for all sites. ECT was administered 3 times weekly for Sites 1, 2, and 3 and 2–3 times weekly for Site 4. Treatment duration was determined based on clinical response and was suspended either upon achieving maximal clinical response defined by either the HDRS or Quick Inventory of Depressive Symptoms or after no appreciable benefit was observed.

All participants provided written informed consent as approved by their local ethical committees or Institutional Review Boards (IRBs), and centralized analysis of pooled data was approved by the Regional Ethic Committee South‐East in Norway (2018/769).

### 2.2 Image processing

Image processing details have previously been outlined (Oltedal et al., 2018). Images were acquired on Siemens 3T (Erlangen, Germany) system for Sites 1, 2 and 3 and a Philips 3T (Gyroscan Intera 3T, Philips Medical Systems, Best, NL) at Site 4. Each site provided pretreatment and post‐treatment 3T T1‐weighted MRI images, with a minimum resolution of 1.3 mm in any direction. Sites uploaded MRIs to a common server for a unified preprocessing approach which included correction for scanner‐specific gradient nonlinearity, registration to a common atlas space, and resampling to a 1 mm isotropic grid. Images were segmented using FreeSurfer version 5.3 and Quarc (Holland & Dale, 2011) was used to estimate regional cortical and subcortical volumes. Table S1 lists the included regions of interest (ROIs).

### 2.3 Symptom dimensions

We previously applied EFA to the pretreatment HDRS‐17 items of this cohort (Wade et al., 2020). Three latent dimensions were identified: somatic disturbances (SoD), core mood and anhedonia (CMA), and insomnia. Symptom dimension change was computed by subtracting its baseline from the post‐treatment score.

### 2.4 Predictive modeling

We used random forest regression (RFR) to predict changes along each symptom dimensions based on patient age, sex, body mass index (BMI), pretreatment symptom dimension severity, primary and secondary electrode placements (patients unresponsive to the initial electrode placement were often transitioned to an alternative electrode configuration), and regional volumetric brain changes. Models were trained and tested using 10‐repeated 10‐fold cross validation in which roughly 90% of the samples were randomly assigned to a training set and the remaining 10% were assigned to a test set. RFR models were fit with 1,000 regression trees and were trained to minimize the root mean squared error (RMSE) of the predicted versus actual symptom change. Prior to model training, two transformations were applied separately to patient data in the training and testing sets to prevent biasing model performance (information leakage). First, to isolate the contributions of regional brain volumes independently apart from any correlated clinical or demographic features, regional volumetric data in the training set was residualized with respect to patient age, sex, BMI, primary and secondary electrode placements, and pretreatment symptom dimension severity. Regression model parameters identified for the training data were used to residualize test set imaging data. Similarly, because multisite imaging data is commonly confounded by scanner‐specific idiosyncrasies, we used an adapted ComBat harmonization algorithm (Fortin et al., 2017; Radua et al., 2020) to harmonize (i.e., mitigate unwanted influences of acquisition site) training and testing imaging data, separately. Harmonization parameters acquired from the training set were applied to the test cohort (see Figure S1).

Model performance was assessed using the sum‐of‐squares $R^{2}$ value (i.e., the fraction of explained variance), $R^{2}\;$= 1 − $\frac{\sum_{i}{\left(y_{i}-{\hat{y}}_{i}\right)}^{2}}{\sum_{i}{\left(y_{i}-\bar{y}\right)}^{2}}$, where $y_{i}$ is the actual symptom change for the *i‐*th subject; ${\hat{y}}_{i}$ is the predicted symptom change for the *i*‐th subject; and $\bar{y}$ is the mean symptom change. The normalized RMSE (NRMSE) value was also reported to facilitate comparisons across scales with different ranges, NRMSE = $\frac{\text{RMSE}}{y_{\max}-y_{\min}}$ where $y_{\max}$ and $y_{\min}$ are the maximum and minimum outcome values, respectively, in the test set; each metric was evaluated across test set predictions (Poldrack, Huckins, & Varoquaux, 2020). The significance of each model's fit was assessed using permutation tests with 100 shuffles. Multiple comparisons made across latent symptom dimensions and benchmarks were jointly adjusted using the false discovery rate method with a significance level of .05. Performance differences for models across symptom dimensions were done using one‐way ANOVAs to test the difference in $R^{2}$ scores resulting from cross validation. The importance of each feature was computed using permutation‐based calculations of the percent increase in mean squared error (PIMSE). This formulation is detailed in Supplementary Methods.

### 2.5 Benchmarks

We compared the performance of models using latent symptom dimensions as outcomes to models using the HDRS‐17 and HDRS‐6 total scores.

### 2.6 Post‐hoc sensitivity analyses

Several sensitivity analyses were conducted to probe model robustness and specificity. First, to directly understand the contribution of neuroimaging features, we evaluated the performance of models that included only regional brain volume predictors, rather than the joint set of volumetric, clinical, and demographic predictors. In this approach, we again regressed the effects of demographic and clinical variables out of the imaging predictors and harmonized them using ComBat.

We secondly explored whether models trained on a specific dimension would be uniquely predictive of the symptom dimension it was trained on or whether it is equally predictive of other dimensions. This would inform potentially unique mechanisms underlying reduction of specific symptom clusters.

Thirdly, we evaluated whether model performance improved when Site 1 participants, where patient symptoms were significantly less improved, were excluded.

We next excluded patients with psychotic features as their symptoms were significantly more reduced than those without psychotic features and were predominantly from Site 2 (see Section 3). However, because psychotic features are known to be indicative of better response to ECT (van Diermen et al., 2018), we kept these patients in the primary analysis.

Lastly, we performed leave‐one‐site out cross validation to evaluate model generalizability to data from sites unseen by the model. Here, however, an additional step was necessary to permit data harmonization. Because these models were each trained on three of the four sites, harmonization parameters for the held‐out site were not available from the training data set. To correct this, an intermediary random forest model was trained to predict site labels of the training observations. This model was then used to predict pseudo‐site labels for the held‐out site. Data from the hold‐out site was then harmonized using these pseudo‐site labels as an approximation.

### 2.7 Code availability

Code for this project is available at https://github.com/bscwade/gemric_latent_symptom_dimension_study.

## 3 RESULTS

### 3.1 Clinical and demographic effects

Patient age differed significantly except for site pairs 1 & 4 and 3 & 4. Neither sex ($\chi^{2}$ = 2.80, df = 3, *p* = .42) nor BMI (*F* = 1.54, *p* > .05) differed significantly across sites. Site 3 had the lowest proportion of patients who underwent right unilateral ECT and the largest number of bitemporal ECT patients. Change in patient HDRS‐17 total score differed across all sites except site pairs 2 & 3 and 3 & 4. Patient HDRS‐17 scores from all sites were reduced more than observed in the healthy control cohort (all *p* < .05, one‐tailed). The degree of symptom reduction did not differ between those with and without a diagnosis of bipolar disorder (*t* = 1.3, df = 9.7, *p* > .05). Symptoms were significantly more reduced among patients with psychotic features compared to those without (*t* = −4.4, df = 20.1, *p* < .001). Detailed patient characteristics are provided in Table 1.

### 3.2 Pretreatment symptom dimensions

As detailed in Wade et al. (2020), the SoD dimension included somatic gastrointestinal (G.I.) symptoms, hypochondriasis, feelings of guilt, genital symptoms, general somatic symptoms, somatic anxiety, psychic anxiety, and psychomotor agitation HDRS items. The CMA dimension was composed of work and interests, weight loss, psychomotor retardation, and depressed mood. The insomnia dimension included early, middle, and late insomnia items (see Figure S2). Notably, suicide and insight items did not adequately load onto a single factor and thus were omitted. The correlated change in symptom dimensions over treatment ranged between .23 and .91 (see Figure S3). The degree of dimension changes also varied by site (Figure S4). All symptom dimensions were significantly reduced across patients relative to controls (all *p* < .01, one‐tailed).

### 3.3 Models including clinical and demographic predictors

**FIGURE 1:**
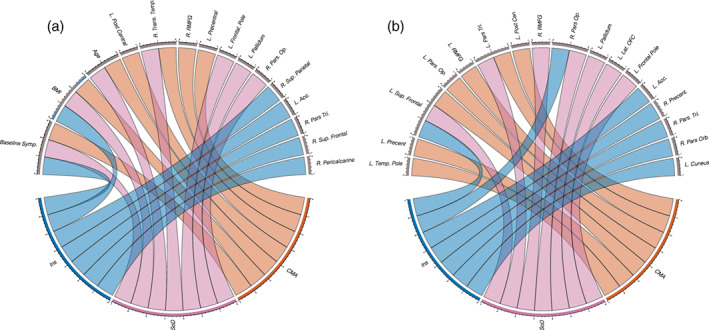
Circular plots highlighting the most important predictors for (a) the full models including patient clinical, demographic, and regional volumetric change predictors and (b) models including only regional volumetric change predictors. Lower halves of the circles represent latent symptom dimensions while upper halves represent important predictors. Color‐coded lines connect symptom dimensions to the predictors that most informed their outcomes

**FIGURE 2:**
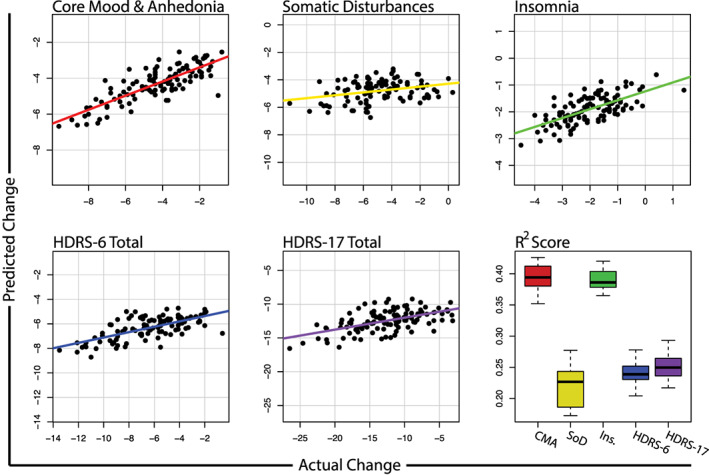
Predicted versus actual change along each latent symptom dimension, HDRS‐6, and HDRS‐17 total scores. Bottom right boxplot shows the distribution of $R^{2}$ scores obtained across repeated cross validation folds

**FIGURE 3:**
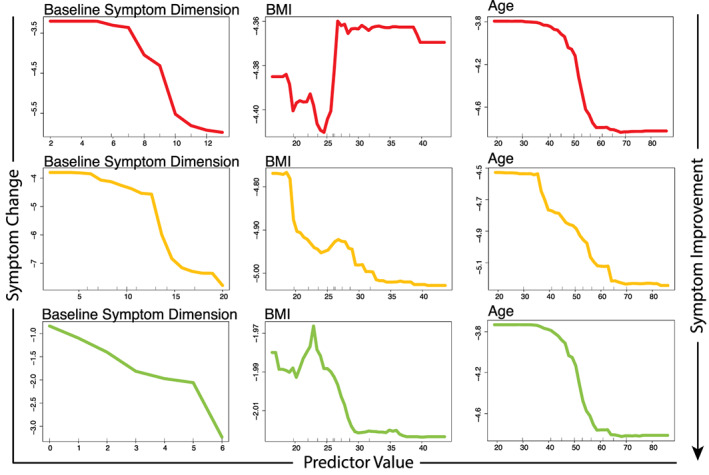
Partial dependence plots illustrating the expected degree of symptom dimension change (*y*‐axis) for observed values of the important clinical or demographic predictors (*x*‐axis) while holding all other model predictors at their observed median values

**FIGURE 4:**
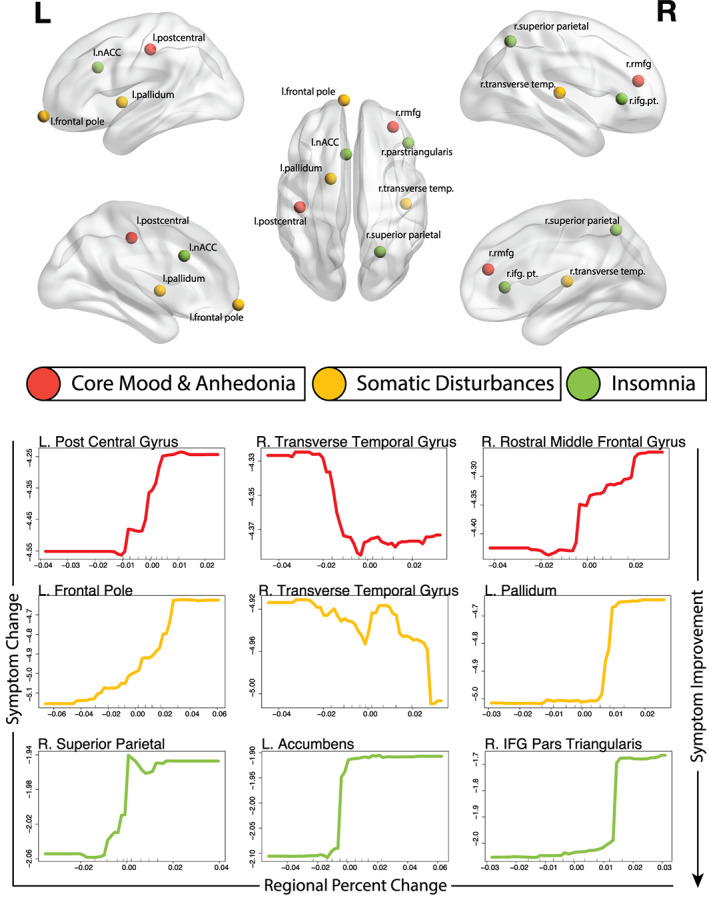
Top: Locations of top three important brain regional change predictors illustrated in Montreal Neurological Institute (MNI) space. Bottom: Partial dependence plots illustrating the expected degree of symptom dimension change (*y*‐axis) for observed values of the important regional volumetric change predictors (*x*‐axis) while holding all other model predictors at their observed median values

The fractional variance explained, $R^{2}$, for the SoD dimension was 22% with an NRMSE of 0.19 (*p* < .01). Model performances are tabulated in Table 2. Here, the most predictive features were baseline symptom dimension severity (PIMSE = 6.4%), BMI (PIMSE = 2.5%), and age (PIMSE = 1.0%). Predictive imaging measures included the left frontal pole, right transverse temporal gyrus, and the left pallidum volumetric changes (all PIMSE <1%). Mappings between important predictors and symptom dimensions are illustrated in Figure 1a and plots of the predicted versus actual change along each symptom dimension is shown in Figure 2. Partial dependence plots (PDP) highlighting the expected marginal change in symptoms for important predictors are illustrated in Figures 3 and 4. Higher reduction of SoD symptoms is expected at higher levels of pretreatment symptoms, BMI, age, and increased transverse temporal gyrus volume change. Conversely, increased volumetric change of the left frontal pole and left pallidum associated with poorer SoD symptom outcomes.

**TABLE 2:** Model performance

Symptom set	Clinical/demographic predictors		No clinical/demographic predictors	
	$R^{2}$	NRMSE	$R^{2}$	NRMSE
SoD	22%	0.19	2.5%	0.21
CMA	39%	0.16	5.6%	0.20
INS	39%	0.15	1.4%	0.19
HDRS‐6	24%	0.21	4.9%	0.24
HDRS‐17	25%	0.21	10%	0.23

Abbreviations: CMA, core mood and anhedonia; INS, insomnia; HDRS‐6, Six‐item Hamilton Depression Rating Scale; HDRS‐17, 17‐item Hamilton Depression Rating Scale; SoD, somatic disturbances.

The CMA dimension $R^{2}$ was 39% with an NRMSE of 0.16 (*p* < .01). The most predictive features included baseline symptom dimension severity (PIMSE = 5.1%), BMI (PIMSE = 2.0%), and age (PIMSE = 0.8%). The most predictive imaging features included the left postcentral gyrus, right transverse temporal gyrus, and right rostral middle frontal gyrus (RMFG) volume changes (all PIMSE <1%). Increased pretreatment CMA symptoms, age, and transverse temporal gyrus volumetric changes were indicative of more reduced CMA symptoms while increased BMI, and volumetric change of the left post central and RMFG associated with poorer CMA outcomes.

Performance for the insomnia dimension was $R^{2}$ of 39% with an NRMSE of 0.15 (*p* < .01). Top predictors for insomnia were baseline symptom severity (PIMSE = 2.6%) and BMI (1.0%). Important imaging features included the right superior parietal lobule, left accumbens, and right pars triangularis volume changes (all PIMSE <1%). Higher pretreatment insomnia symptoms, BMI, age, and volumetric reductions of the right superior parietal lobule, left accumbens, and pars triangularis associated with more reduced insomnia symptoms.

### 3.4 Models excluding clinical and demographic predictors

Model performance was substantially lower when clinical and demographic features were not used though all were predicted significantly above chance levels. The $R^{2}$ score for the SoD dimension was 2.5% while the NRMSE was 0.21 (both *p* < .05). Important predictors for these models are reported in Supplementary Results, illustrated in Figure 1b, and Figure S5 illustrates PDP plots for these models. The CMA dimension $R^{2}$ score was reduced to 5.6% with an NRMSE of 0.20 (both *p* < .05). The insomnia symptom $R^{2}$ score lowered to 1.4% with an NRMSE of 0.19 (both *p* < .05). Here, CMA dimension change was predicted significantly more accurately than SoD and insomnia symptoms (all *p* < .01).

### 3.5 Benchmark predictions

When clinical and demographic predictors were included, $R^{2}$ scores for the prediction of HDRS‐6 change was 24% with an NRMSE of 0.21 (*p* < .01). The $R^{2}$ score for the HDRS‐17 change was 25% with an NRMSE of 0.21 (*p* < .01). Important features for benchmark predictors are outlined in Supplementary Results. The CMA and insomnia dimensions were predicted significantly more accurately than the HDRS‐6, HDRS‐17, and SoD dimension (*p* < .0001); the performance of SoD and HDRS‐6 and HDRS‐17 models did not significantly differ (*p* > .05). In models that excluded clinical and demographic predictors and instead used only neuroimaging predictors, the HDRS‐6 $R^{2}$ score was 4.9% with an NRMSE of 0.24 while the HDRS‐17 $R^{2}$ was 10% with an NRMSE of 0.23 (all *p* < .05). With clinical and demographic measures excluded, the HDRS‐17 change was predicted significantly more accurately than the CMA, SoD, insomnia, and HDRS‐6 symptom sets (all *p* < .0001).

### 3.6 Cross factor predictions and model specificity

Model performance was lower, on average, when models were trained and tested on separate symptom dimensions. However, models trained on CMA symptoms and used to predict SoD symptoms, or vice versa, did not significantly differ in distributions of $R^{2}$ scores from models trained and tested within these respective dimensions.

### 3.7 Models excluding Site 1

Models excluding patients from Site 1 and including clinical and demographic predictors performed higher, on average with $R^{2}$ scores of 35%, 38%, and 47% for SoD, CMA, and insomnia dimensions, respectively. $R^{2}$ scores were 22% and 28% for the HDRS‐6 and HDRS‐17 benchmarks, respectively. When these models excluded clinical and demographic predictors, however, all $R^{2}$ scores were below zero.

### 3.8 Leave‐one‐site‐out cross validation

When clinical and demographic predictors were included, all $R^{2}$ scores were negative under leave‐one‐site‐out cross validation (LOOCV) except for the insomnia dimension ($R^{2}$ = 30%). Exclusion of clinical and demographic predictors produced negative $R^{2}$ scores for all symptom dimensions.

When patients from Site 1 were excluded, LOOCV models were generally improved with $R^{2}$ scores of 6.2%, 24%, 52%, −10%, and 3% for CMA, SoD, insomnia, HDRS‐6, and HDRS‐17 symptoms, respectively; however, dropping pretreatment symptoms lowered all $R^{2}$ scores below zero except for the insomnia dimension ($R^{2}$ = 1.9%).

## 4 DISCUSSION

Our study extends recent work using exploratory factor analysis to identify three homogenous latent symptom dimensions of treatment‐resistant MDD. We used data‐driven methods to identify clinical, demographic, and distributed patterns of regional volumetric brain changes associated with ECT‐related recovery along these latent symptom dimensions of depression. Models predicting recovery along core mood and anhedonia and insomnia dimensions were predicted significantly more accurately than the more widely used HDRS‐6 and HDRS‐17 total scores. Further, treatment‐related recovery along these symptom dimensions was associated with unique and overlapping sets of clinical, demographic, and volumetric brain regions. Brain regions informative of symptom changes were generally well‐aligned with existing literature.

Easily‐acquired and inexpensive clinical and demographic measures including pretreatment symptom severity, patient age, and body mass index (BMI) were generally the most informative predictors of change. Previous studies have reported that increased symptom severity and presence of psychotic features are indicative of better response to ECT (van Diermen et al., 2018). Increased patient age has widely been linked with better ECT responsivity (Kranaster et al., 2018; O'Connor et al., 2001). However, the etiology of depression in elderly patients more frequently involves cardiovascular pathology, cognitive impairment, or chronic medical illness (Alexopoulos, 2005), which in addition to normal aging effects of brain tissue loss, could impact response to ECT. In our sample, patient age differed significantly by site that may have limited our model generalizability to new sites. Earlier work has also reported that elevated BMI is associated with reduced white matter integrity (Repple et al., 2018), gray matter reductions of frontal regions, and a more chronic course of depression (Opel et al., 2015). Interestingly, while elevated BMI predicted poorer outcomes for the CMA symptom dimension, it predicted better treatment response for the SoD and insomnia symptom clusters. This effect is likely driven by the inclusion of the HDRS weight loss item in the CMA factor. Here, we observed that, relative to patients who exhibited no change in the weight loss item, those who reduced their weight loss symptoms had a significantly lower pretreatment BMI (*p* < .05).

Although clinical and demographic measures were the most informative predictors of outcomes, models using only changes in regional brain volumes as predictors were highly significant and explained between 1 and 10% of outcome variability across symptom dimensions and remain valuable. Regional volumetric predictors of change along the CMA dimension included the left postcentral gyrus, right transverse temporal gyrus, and right rostral middle frontal gyrus (RMFG). Earlier studies have identified ECT‐related volumetric changes in the left postcentral gyrus (Jiang et al., 2018), and rostral middle frontal gyrus (Mulders et al., 2020), while other studies have identified the pretreatment middle frontal gyrus structure (Jiang et al., 2018) as a predictor of ECT response. Several temporal regions have been reported to change over ECT index (Ota et al., 2015; van Eijndhoven et al., 2016). As regional volumetric changes are correlated with the local magnitude of the electric field (Argyelan et al., 2019), volumetric changes in the postcentral gyrus and transverse temporal lobes are expected as they are proximal to electrodes in RUL ECT.

Imaging predictors of SoD symptom reduction included volumetric changes in the left frontal pole, right transverse temporal gyrus, and the left pallidum. The transverse temporal gyrus was also a predictor of CMA change. As SoD and CMA symptom changes were highly correlated and the temporal gyrus is proximal to ECT electrodes, this is perhaps unsurprising but may also suggest a common neurobiological basis for these symptoms. Volumetric increases of the right frontal pole was likewise inversely related to antidepressant response in a related cohort using the Montgomery‐Asberg Depression Rating Scale and HDRS total scores as outcomes (Mulders et al., 2020). Similarly, an earlier study of our own in a partially overlapping cohort identified that volumetric increases in the right pallidum were associated with poorer ECT response (Wade et al., 2016); here, we report the same trend for the contralateral pallidal volume.

Volumetric changes of the right superior parietal lobule, left accumbens, and right pars triangularis were predictive of change in the insomnia dimension. Although their specific relationships to symptoms of insomnia are difficult to interpret, ECT‐related changes in the accumbens have previously been related to antidepressant response (Wade et al., 2016). The superior parietal lobule is a component of the default‐mode network (DMN) which may indirectly modulate emotional processes underlying depressive symptoms (Zhang, Peng, Sweeney, Jia, & Gong, 2018); this structure also neighbors the supramarginal gyrus which has been shown to relate to ECT response (Mulders et al., 2020).

The imaging measures informative of treatment‐related symptom changes are biologically plausible. Despite differences in analysis methods and samples, findings also appear collectively aligned with previous reports of treatment‐predictive and treatment‐responsive biomarkers of ECT response, which point to the involvement of somatomotor cortex (Leaver et al., 2018), RMFG (Mulders et al., 2020), and striatum and basal ganglia (Wade et al., 2016). Notably, the current findings expand on previous work by shedding new light on how these broad neural systems along with patient clinical/demographic characteristics relate to more specific symptom constellations.

Importantly, because heterogeneity was a factor not only at the level of symptoms but also across sites, we decided to conduct leave‐one‐site‐out cross validation (LOOCV) to evaluate how generalizable models were across sites. Here, model performances were widely reduced to chance levels. Because patients from Site 1 were significantly less responsive to ECT, we evaluated LOOCV models when these patients were excluded. Here, models generalized well across sites, highlighting that model extrapolation to outcome ranges outside of those in which the model was trained remains challenging, though recent advancements may be of use in future studies (Wachinger & Reuter, 2016; Zhang, Nettleton, & Zhu, 2019). This further underscores the importance of considering heterogeneity of treatment protocols in naturalistic multisite studies as this is a demonstrable source of variance. Though model performance was improved for both the primary analysis and under LOOCV, we chose to retain patients from Site 1 in the main analysis in order to evaluate the performance of these models across the natural variety of ECT protocols observed across the world. Clearly, however, models can be refined by ensuring outcomes are similar across sites.

### 4.1 Limitations

Naturalistic treatment protocols across this multisite study are a potential limitation; however, this design also builds the natural diversity of ECT treatment protocols and outcomes seen worldwide into our models. Variation in treatment parameters was naturally confounded with acquisition site. We did, however, include both site and electrode placement as predictors though electrode placement was not an important predictor. Relatedly, patient inclusion/exclusion criteria varied by site though all had in common that patients were required to have failed at least one prior treatment or that patients immediately needed ECT. A minority of patients were included with diagnoses of bipolar disorder and depression with psychotic features. Symptom changes did not significantly differ among those with bipolar disorder but were significantly more reduced among those with psychotic features as is commonly observed (van Diermen et al., 2018). Site 1 patients were also least responsive with an average HDRS‐17 reduction of 3.8 points. These patients were tapered off of antidepressant medication prior to ECT index, though concurrent medications do not reliably effect the efficacy of ECT (Fink, 1994; Haskett & Loo, 2010). Exclusion of patients from Site 1 largely improved model performance and generalizability but this gain is counterbalanced against the benefit of training these models using the range of ECT outcomes seen around the world. Lastly, we emphasize that prospective prediction of clinical outcomes was not attempted; this would require exclusive use of pretreatment predictors.

### 4.2 Conclusions and future directions

Taken together, our findings provide new evidence that use of homogenized latent symptom dimensions of multi‐item scales can improve the detection of imaging, demographic, and clinical biomarkers related to the trajectories of specific symptom constellations. While clinical and demographic measures accounted for more outcome variability, neuroimaging measures of regions often implicated in the pathology of depression and ECT‐related treatment response were significantly predictive and accounted for between 1 and 10% of outcome variability when used alone. As neurostimulation methods become more refined and capable of targeting more specific neural systems, it is plausible that findings such as these will inform the targeting of neural systems underlying more specific symptom dimensions. Future work will explore whether prospective prediction of change in these symptom dimensions will similarly be predicted more accurately than more heterogenous total scores.

## CONFLICT OF INTEREST

The authors declare no conflicts of interest relevant to this work.

## Supporting information


**Appendix S1**: Supplementary Methods

**Supplementary Figure S1** Outline of the cross‐validation procedure used for the main analysis. At each fold, relevant clinical and demographic measures were regressed out of the volumetric training data and the parameters from that model were applied to residualize volumetric data in the test set. Similarly, ComBat harmonization was used to minimize effect of scanners in the volumetric data within the training cohort. ComBat parameters discovered in the training data were used to harmonize volumetric test data. Following these transformations, the random forest regression model was fit to the training data. The fitted model was used to predict symptom change in the test data and the process was repeated

**Supplementary Figure S2** The three‐factor solution of the pretreatment Hamilton Depression Rating Scale identified in our previous study and used here

**Supplementary Figure S3** Paired correlation of change across all latent symptom dimensions and the HDRS‐17 and HDRS‐6 total scores over treatment

**Supplementary Figure S4** Differential degrees of change for symptom dimensions across each site

**Supplementary Figure S5** Outline of model using only regional volumetric changes as predictors. Top: Locations of top three important brain regional change predictors illustrated in Montreal Neurological Institute (MNI) space. Bottom: Partial dependence plots illustrating the expected degree of symptom dimension change (*y*‐axis) for observed values of the important regional volumetric change predictors (*x*‐axis) while holding all other model predictors at their observed median values

**Supplementary Table S1** Bilateral regions included as predictors

## Data Availability

The data that support the findings of this study are restricted to members of The Global ECT‐MRI Research Collaboration due to privacy and ethical concerns.

## References

[hbm25620-bib-0001] Alexopoulos, G. S. (2005). Depression in the elderly. Lancet, 365, 1961–1970. 15936426 10.1016/S0140-6736(05)66665-2

[hbm25620-bib-0002] Argyelan, M. , Oltedal, L. , Deng, Z.‐D. , Wade, B. , Bikson, M. , Joanlanne, A. , … Abbott, C. (2019). Electric field causes volumetric changes in the human brain. eLife, 8. 10.7554/eLife.49115. PMC6874416 31644424

[hbm25620-bib-0003] Bech, P. , Allerup, P. , Gram, L. F. , Reisby, N. , Rosenberg, R. , Jacobsen, O. , & Nagy, A. (1981). The Hamilton Depression Scale. Acta Psychiatrica Scandinavica, 63, 290–299. 7015793 10.1111/j.1600-0447.1981.tb00676.x

[hbm25620-bib-0004] Faries, D. , Herrera, J. , Rayamajhi, J. , Debrota, D. , Demitrack, M. , & Potter, W. Z. (2000). The responsiveness of the Hamilton Depression Rating Scale. Journal of Psychiatric Research, 34, 3–10. 10696827 10.1016/s0022-3956(99)00037-0

[hbm25620-bib-0005] Fink, M. (1994). Combining electroconvulsive therapy and drugs. CNS Drugs, 1, 370–376. 10.2165/00023210-199401050-00007

[hbm25620-bib-0006] Fortin, J. P. , Parker, D. , Tunç, B. , Watanabe, T. , Elliott, M. A. , Ruparel, K. , … Shinohara, R. T. (2017). Harmonization of multi‐site diffusion tensor imaging data. NeuroImage, 161, 149–170. 10.1016/j.neuroimage.2017.08.047 28826946 PMC5736019

[hbm25620-bib-0007] Haq, A. U. , Sitzmann, A. F. , Goldman, M. L. , Maixner, D. F. , & Mickey, B. J. (2015). Response of depression to electroconvulsive therapy: A meta‐analysis of clinical predictors. The Journal of Clinical Psychiatry, 76, 1374–1384. 26528644 10.4088/JCP.14r09528

[hbm25620-bib-0008] Haskett, R. F. , & Loo, C. (2010). Adjunctive psychotropic medications during electroconvulsive therapy in the treatment of depression, mania, and schizophrenia. The Journal of ECT, 26, 196–201. https://pubmed.ncbi.nlm.nih.gov/20805728 20805728 10.1097/YCT.0b013e3181eee13f PMC2952444

[hbm25620-bib-0009] Holland, D. , & Dale, A. M. (2011). Nonlinear registration of longitudinal images and measurement of change in regions of interest. Medical Image Analysis, 15, 489–497. 10.1016/j.media.2011.02.005 21388857 PMC3115407

[hbm25620-bib-0010] James, S. L. , Abate, D. , Abate, K. H. , Abay, S. M. , Abbafati, C. , Abbasi, N. , … Murray, C. J. L. (2018). Global, regional, and national incidence, prevalence, and years lived with disability for 354 diseases and injuries for 195 countries and territories, 1990–2017: A systematic analysis for the Global Burden of Disease Study 2017. Lancet, 392, 1789–1858. 30496104 10.1016/S0140-6736(18)32279-7 PMC6227754

[hbm25620-bib-0011] Jiang, R. , Abbott, C. C. , Jiang, T. , Du, Y. , Espinoza, R. , Narr, K. L. , … Calhoun, V. D. (2018). SMRI biomarkers predict electroconvulsive treatment outcomes: Accuracy with independent data sets. Neuropsychopharmacology, 43, 1078–1087. 28758644 10.1038/npp.2017.165 PMC5854791

[hbm25620-bib-0012] Kranaster, L. , Aksay, S. S. , Bumb, J. M. , Hoyer, C. , Jennen‐Steinmetz, C. , & Sartorius, A. (2018). A novel seizure quality index based on ictal parameters for optimizing clinical decision making in electroconvulsive therapy. Part 1: Development. European Archives of Psychiatry and Clinical Neuroscience, 268, 819–830. 29876649 10.1007/s00406-018-0910-6

[hbm25620-bib-0013] Leaver, A. M. , Wade, B. , Vasavada, M. , Hellemann, G. , Joshi, S. H. , Espinoza, R. , & Narr, K. L. (2018). Fronto‐temporal connectivity predicts ECT outcome in major depression. Frontiers in Psychiatry, 9. 10.3389/fpsyt.2018.00092 PMC5871748 29618992

[hbm25620-bib-0014] Michael Bagby, R. , Ryder, A. G. , Deborah Schuller, M. R. , & Marshall, M. B. (2004). The Hamilton Depression Rating Scale: Has the gold standard become a lead weight? American Journal of Psychiatry, 161(12), 2163–2177. http://ajp.psychiatryonline.org 10.1176/appi.ajp.161.12.2163 15569884

[hbm25620-bib-0015] Mulders, P. C. R. , Llera, A. , Beckmann, C. F. , Vandenbulcke, M. , Stek, M. , Sienaert, P. , … Tendolkar, I. (2020). Brain stimulation structural changes induced by electroconvulsive therapy are associated with clinical outcome. Brain Stimulation, 13, 696–704. 10.1016/j.brs.2020.02.020 32289700

[hbm25620-bib-0016] O'Connor, M. K. , Knapp, R. , Husain, M. , Rummans, T. A. , Petrides, G. , Smith, G. , … Kellner, C. (2001). The influence of age on the response of major depression to electroconvulsive therapy: A C.O.R.E. report. The American Journal of Geriatric Psychiatry, 9, 382–390. 11739064

[hbm25620-bib-0017] Oltedal, L. , Narr, K. L. , Abbott, C. , Anand, A. , Argyelan, M. , Bartsch, H. , … Dale, A. M. (2018). Volume of the human hippocampus and clinical response following electroconvulsive therapy. Biological Psychiatry, 84, 574–581. 10.1016/j.biopsych.2018.05.017 30006199 PMC6697556

[hbm25620-bib-0018] Opel, N. , Redlich, R. , Grotegerd, D. , Dohm, K. , Heindel, W. , Kugel, H. , … Dannlowski, U. (2015). Obesity and major depression: Body‐mass index (BMI) is associated with a severe course of disease and specific neurostructural alterations. Psychoneuroendocrinology, 51, 219–226. 25462895 10.1016/j.psyneuen.2014.10.001

[hbm25620-bib-0019] Ota, M. , Noda, T. , Sato, N. , Okazaki, M. , Ishikawa, M. , Hattori, K. , … Kunugi, H. (2015). Effect of electroconvulsive therapy on gray matter volume in major depressive disorder. Journal of Affective Disorders, 186, 186–191. 26247910 10.1016/j.jad.2015.06.051

[hbm25620-bib-0020] Ousdal, O. T. , Argyelan, M. , Narr, K. L. , Abbott, C. , Wade, B. , Vandenbulcke, M. , … Oltedal, L. (2020). Brain changes induced by electroconvulsive therapy are broadly distributed. Biological Psychiatry, 87, 451–461. 31561859 10.1016/j.biopsych.2019.07.010

[hbm25620-bib-0021] Poldrack, R. A. , Huckins, G. , & Varoquaux, G. (2020). Establishment of best practices for evidence for prediction: A review. JAMA Psychiatry, 77, 534–540. 31774490 10.1001/jamapsychiatry.2019.3671 PMC7250718

[hbm25620-bib-0022] Radua, J. , Vieta, E. , Shinohara, R. , Kochunov, P. , Quidé, Y. , Green, M. J. , … van Erp, T. (2020). Increased power by harmonizing structural MRI site differences with the ComBat batch adjustment method in ENIGMA. NeuroImage, 218, 116956. 32470572 10.1016/j.neuroimage.2020.116956 PMC7524039

[hbm25620-bib-0023] Repple, J. , Opel, N. , Meinert, S. , Redlich, R. , Hahn, T. , Winter, N. R. , … Dannlowski, U. (2018). Elevated body‐mass index is associated with reduced white matter integrity in two large independent cohorts. Psychoneuroendocrinology, 91, 179–185. 29571075 10.1016/j.psyneuen.2018.03.007

[hbm25620-bib-0024] Schmitgen, M. M. , Kubera, K. M. , Depping, M. S. , Nolte, H. M. , Hirjak, D. , Hofer, S. , … Wolf, R. C. (2020). Exploring cortical predictors of clinical response to electroconvulsive therapy in major depression. European Archives of Psychiatry and Clinical Neuroscience, 270, 253–261. 31278421 10.1007/s00406-019-01033-w

[hbm25620-bib-0025] Tokutsu, Y. , Umene‐Nakano, W. , Shinkai, T. , Yoshimura, R. , Okamoto, T. , Katsuki, A. , … Nakamura, J. (2013). Follow‐up study on electroconvulsive therapy in treatment‐resistant depressed patients after remission: A chart review. Clinical Psychopharmacology and Neuroscience, 11, 34–38. 23678353 10.9758/cpn.2013.11.1.34 PMC3650296

[hbm25620-bib-0026] Trivedi, M. H. , Rush, A. J. , Wisniewski, S. R. , Nierenberg, A. A. , Warden, D. , Ritz, L. , … Fava, M. (2006). Evaluation of outcomes with citalopram for depression using measurement‐based care in STAR*D: Implications for clinical practice. The American Journal of Psychiatry, 163, 28–40. 16390886 10.1176/appi.ajp.163.1.28

[hbm25620-bib-0027] van Diermen, L. , van den Ameele, S. , Kamperman, A. M. , Sabbe, B. C. G. , Vermeulen, T. , Schrijvers, D. , & Birkenhäger, T. K. (2018). Prediction of electroconvulsive therapy response and remission in major depression: Meta‐analysis. The British Journal of Psychiatry, 212, 71–80. https://www.cambridge.org/core/article/prediction-of-electroconvulsive-therapy-response-and-remission-in-major-depression-metaanalysis/259FD7600E652E9D272481FC6D87F4F9 29436330 10.1192/bjp.2017.28

[hbm25620-bib-0028] van Eijndhoven, P. , Mulders, P. , Kwekkeboom, L. , van Oostrom, I. , van Beek, M. , Janzing, J. , … Tendolkar, I. (2016). Bilateral ECT induces bilateral increases in regional cortical thickness. Translational Psychiatry, 6, e874–e874. https://pubmed.ncbi.nlm.nih.gov/27552587 27552587 10.1038/tp.2016.139 PMC5022085

[hbm25620-bib-0029] van Loo, H. M. , de Jonge, P. , Romeijn, J. W. , Kessler, R. C. , & Schoevers, R. A. (2012). Data‐driven subtypes of major depressive disorder: A systematic review. BMC Medicine, 10. 10.1186/1741-7015-10-156. PMC3566979 23210727

[hbm25620-bib-0030] Wachinger, C. , & Reuter, M. (2016). Domain adaptation for Alzheimer's disease diagnostics. NeuroImage, 139, 470–479. 27262241 10.1016/j.neuroimage.2016.05.053 PMC4983466

[hbm25620-bib-0031] Wade, B. S. C. , Hellemann, G. , Espinoza, R. T. , Woods, R. P. , Joshi, S. H. , Redlich, R. , … Narr, K. L. (2020). Depressive symptom dimensions in treatment‐resistant major depression and their modulation with electroconvulsive therapy. The Journal of ECT, 36, 123–129. https://journals.lww.com/ectjournal/Fulltext/2020/06000/Depressive_Symptom_Dimensions_in.10.aspx 31464814 10.1097/YCT.0000000000000623 PMC7044066

[hbm25620-bib-0032] Wade, B. S. C. , Joshi, S. H. , Njau, S. , Leaver, A. M. , Vasavada, M. , Woods, R. P. , … Narr, K. L. (2016). Effect of electroconvulsive therapy on striatal morphometry in major depressive disorder. Neuropsychopharmacology, 41, 2481–2491. 27067127 10.1038/npp.2016.48 PMC4987846

[hbm25620-bib-0033] Williams, J. B. W. (2001). Standardizing the Hamilton Depression Rating Scale: Past, present, and future. European Archives of Psychiatry and Clinical Neuroscience, 251, 6–12. 11824839 10.1007/BF03035120

[hbm25620-bib-0034] Zhang, F.‐F. , Peng, W. , Sweeney, J. A. , Jia, Z.‐Y. , & Gong, Q.‐Y. (2018). Brain structure alterations in depression: Psychoradiological evidence. CNS Neuroscience & Therapeutics, 24, 994–1003. https://pubmed.ncbi.nlm.nih.gov/29508560 29508560 10.1111/cns.12835 PMC6489983

[hbm25620-bib-0035] Zhang, H. , Nettleton, D. , & Zhu, Z. (2019). Regression‐enhanced random forests. arXiv.

