# Supplementary material for: Accounting for symptom heterogeneity can improve neuroimaging models of antidepressant response after electroconvulsive therapy
Source: Hum Brain Mapp. 2021 Aug 13;42(16):5322–33. doi: 10.1002/hbm.25620 (PMC8519875; doi:10.1002/hbm.25620)
Supplement: Supplementary file 1 — Appendix S1: Supplementary Methods [file HBM-42-5322-s006.docx]

**Supplementary Methods**

*Feature Importance*

The importance of individual features in this multivariate framework to the prediction of symptom dimension change was calculated using the random forest permutation importance (19). In short, individual regression trees in random forests are constructed using a bootstrapped sample of the original dataset. Roughly one-third of the sample is excluded from individual trees; this held-out third is referred to as an out-of-bag (OOB) sample and is used to monitor model performance and to calculate individual feature importance. Once a tree has been constructed, values of individual predictors in the OOB data are randomly permuted. The OOB observations are then passed through the given tree and the tree’s performance is recorded both for permuted and true-labeled data. The difference between the permuted and non-permuted OOB performance is averaged across all trees in the forest to provide an unbiased estimate of feature importance. Here, this measure is the percent increase in mean squared error (PIMSE) of the OOB predictions.

**Supplementary Results**

*Models Excluding Clinical & Demographic Predictors*

Model performance was substantially lower when clinical and demographic features were not used. For the SoD dimension, the fraction of explained variance, $R^{2}$, was 2.5% while the NRMSE of the prediction was 0.21 (both p<0.05). The most important features in the SoD model (i.e., those with the largest PIMSE values) included the right rostral middle frontal gyrus (PIMSE=0.22%), right pars opercularis (PIMSE=0.20), and the left pallidum (PIMSE=0.19) volume changes; see **Figure 2(b)**. **Figure 5** illustrates PDP plots for important predictors in models excluding non-imaging predictors.

For the CMA dimension, $R^{2}$ was 5.6% while the NRMSE was 0.20 (both p<0.05). The most informative features for change along the CMA dimension included the left temporal pole (PIMSE=0.37%), left precentral gyrus (PIMSE=0.26%), and the left superior frontal gyrus (PIMSE=0.23%) volume changes.

The fractional variance explained for change in the insomnia dimension was 1.4% with an NRMSE of 0.19 (both p<0.05). The most predictive features included the left accumbens area (PIMSE=0.06%), the right precentral gyrus (PIMSE=0.05%), and the right pars triangularis (PIMSE=0.05%) volume changes.

*Benchmark Predictions*

When clinical and demographic predictors were included, the $R^{2}$ for the prediction of HDRS-6 change was 24% with an NRMSE of 0.21 (p<0.01). The top predictors were again baseline symptom severity (PIMSE=4.5%), age (PIMSE=3.2%), and BMI (PIMSE=1.7%). Top imaging features were the right transverse temporal gyrus, left precentral, and left superior frontal gyrus volume changes (all PIMSE $\leq$1%).

The $R^{2}$ score for prediction of HDRS-17 change was 25% with an NRMSE of 0.21 (p<0.01). The most informative features were baseline symptom severity (PIMSE=16%), BMI (6.7%), and age (4.4%). Top imaging predictors included the left frontal pole, right transverse temporal gyrus, and the left hippocampus volumetric change (all PIMSE <2%).

The CMA and insomnia dimensions were predicted significantly more accurately than the HDRS-6, HDRS-17, and SoD dimension (p<0. 0001); however, the performance of SoD and HDRS-6 and HDRS-17 models did not significantly differ (p>0.05).

With clinical and demographic predictors excluded, the HDRS-6 $R^{2}$ score was 4.9% with an NRMSE of 0.24 while the HDRS-17 $R^{2}$ was 10% with an NRMSE of 0.23 (all p<0.05). The most predictive features for HDRS-6 change included the left superior frontal gyrus, left precentral gyrus, and right rostral middle frontal gyrus volume changes (all PIMSE <1%). Top predictors for HDRS-17 change included the right pars opercularis, left superior frontal gyrus, and left pars triangularis volume changes (all PIMSE <2%).

*Models Excluding Patients with Psychotic Features*

Excluding patients with psychotic features generally reduced model performance. $R^{2}$ scores for models including clinical, demographic, and volumetric predictors were 25%, 2.7%, and 35% for the SoD, CMA, and insomnia dimensions, respectively. $R^{2}$ scores were 11 % and 10% for the HDRS-6 and HDRS-17 total score predictions.

$R^{2}$ scores using only volumetric predictors were 1.4%, 3.0%, and 2.7% for the SoD, CMA, and insomnia dimensions, respectively, while $R^{2}$ scores were 1.6%, and 5.9% for the HDRS-6 and HDRS-17 total score predictions.
