# Supplementary material for: Accounting for symptom heterogeneity can improve neuroimaging models of antidepressant response after electroconvulsive therapy
Source: Hum Brain Mapp. 2021 Aug 13;42(16):5322–33. doi: 10.1002/hbm.25620 (PMC8519875; doi:10.1002/hbm.25620)
Supplement: Supplementary file 2 — Supplementary Figure S1 Outline of the cross‐validation procedure used for the main analysis. At each fold, relevant clinical and demographic measures were regressed out of the volumetric training data and the parameters from that model were applied to residualize volumetric data in the test set. Similarly, ComBat harmonization was used to minimize effect of scanners in the volumetric data within the training cohort. ComBat parameters discovered in the training data were used to harmonize volumetric test data. Following these transformations, the random forest regression model was fit to the training data. The fitted model was used to predict symptom change in the test data and the process was repeated [file HBM-42-5322-s004.pdf]

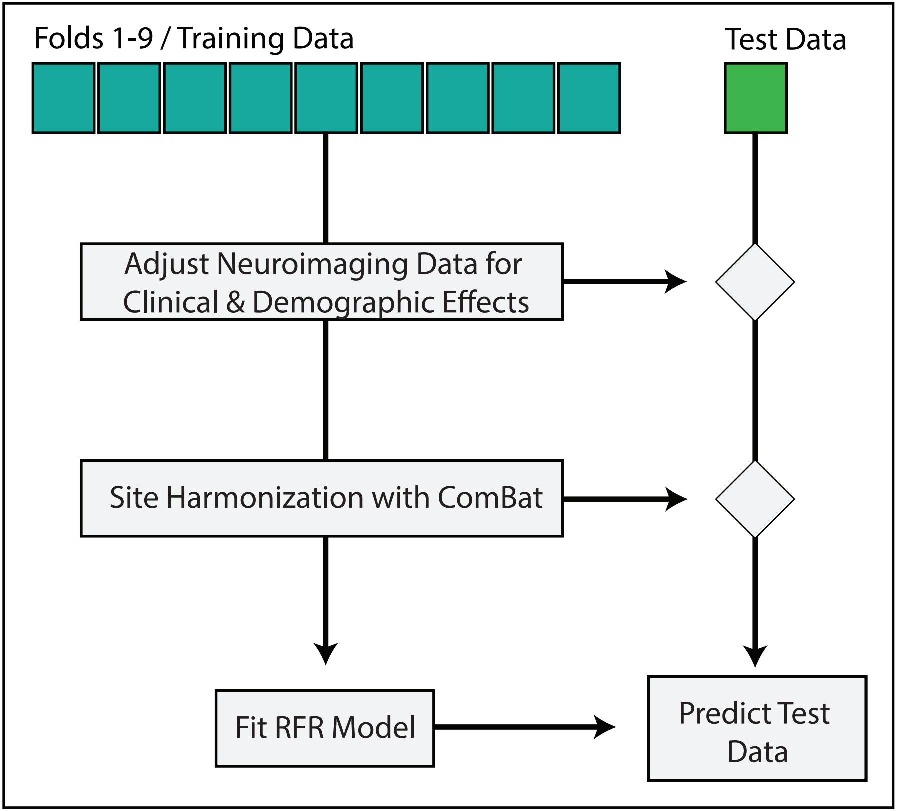
Folds 1-9 / Training Data
Test Data
Adjust Neuroimaging Data for Clinical & Demographic Effects
Site Harmonization with ComBat
Fit RFR Model
Predict Test Data
