# Supplementary figures and images for: Accounting for symptom heterogeneity can improve neuroimaging models of antidepressant response after electroconvulsive therapy
Source: Hum Brain Mapp. 2021 Aug 13;42(16):5322–33. doi: 10.1002/hbm.25620 (PMC8519875; doi:10.1002/hbm.25620)

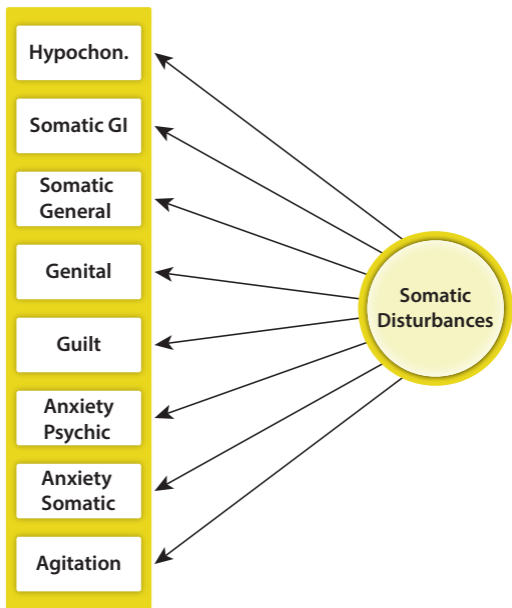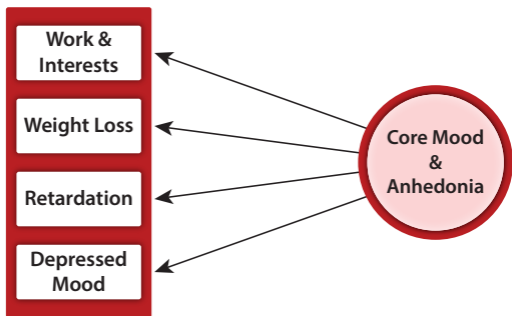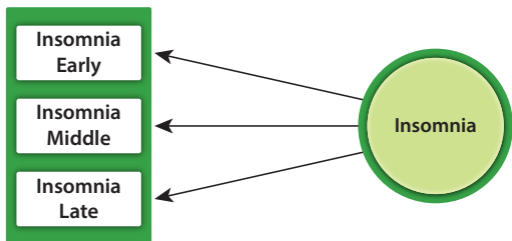

Supplement: Supplementary file 3 — Supplementary Figure S2 The three‐factor solution of the pretreatment Hamilton Depression Rating Scale identified in our previous study and used here [file HBM-42-5322-s003.pdf]

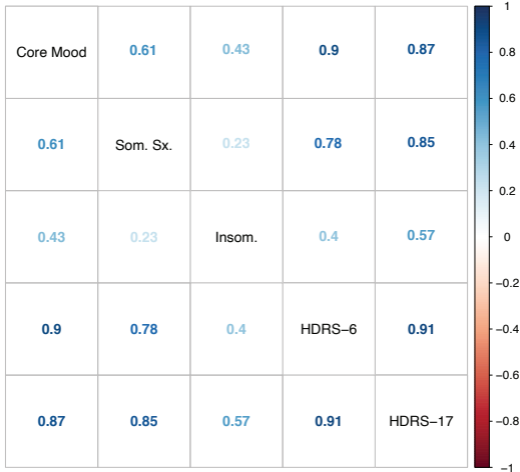

Supplement: Supplementary file 4 — Supplementary Figure S3 Paired correlation of change across all latent symptom dimensions and the HDRS‐17 and HDRS‐6 total scores over treatment [file HBM-42-5322-s007.pdf]

Symptom Dimension Change

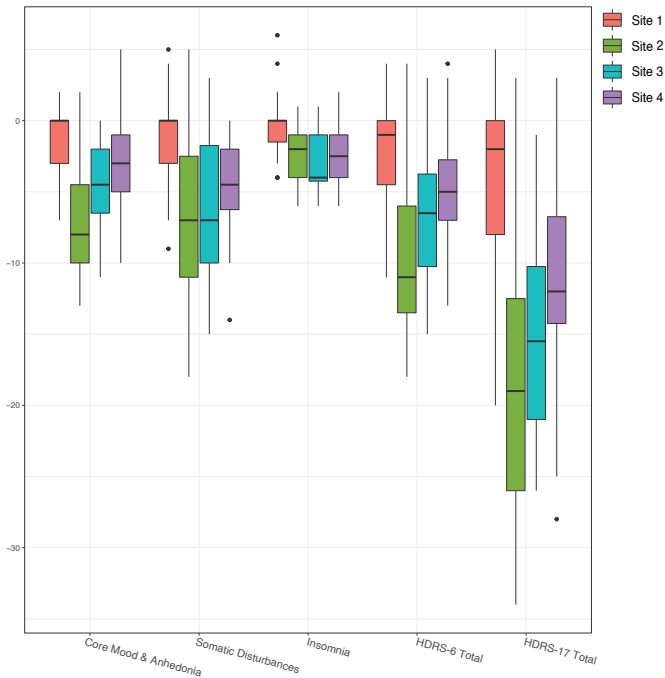

Supplement: Supplementary file 5 — Supplementary Figure S4 Differential degrees of change for symptom dimensions across each site [file HBM-42-5322-s001.pdf]

L

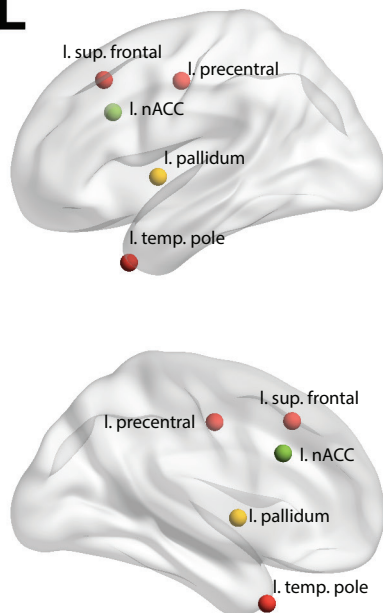

R

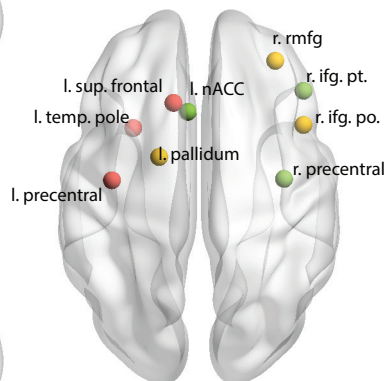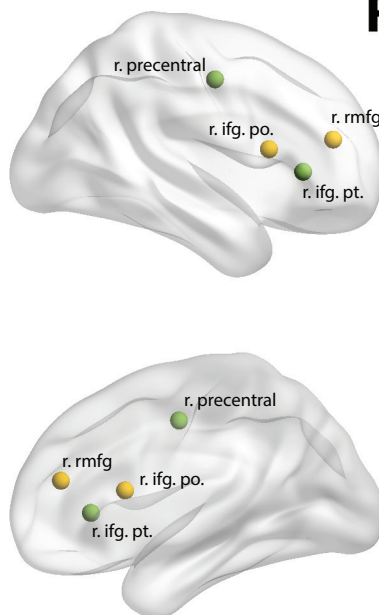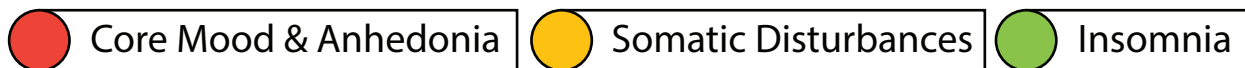

Symptom Change

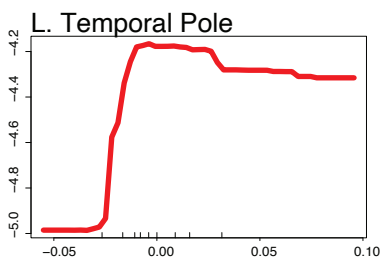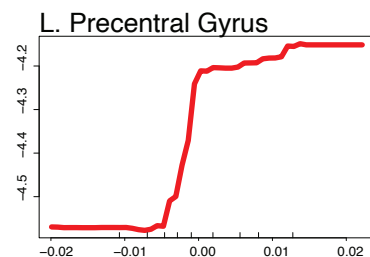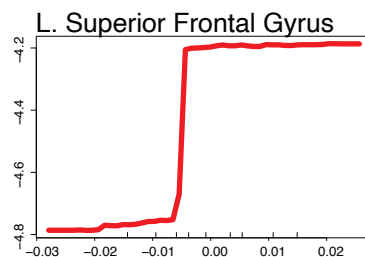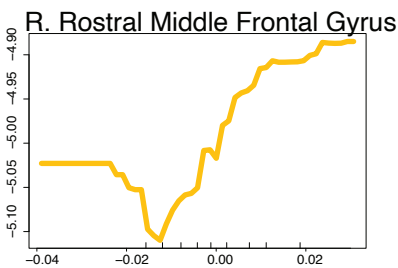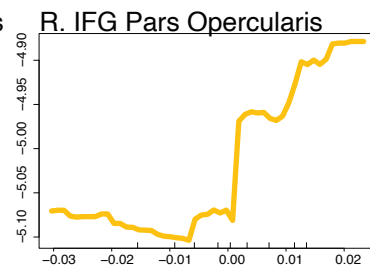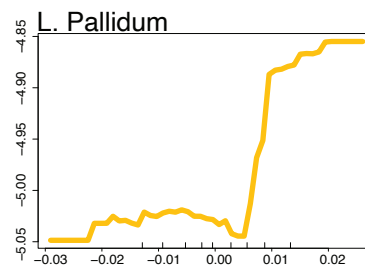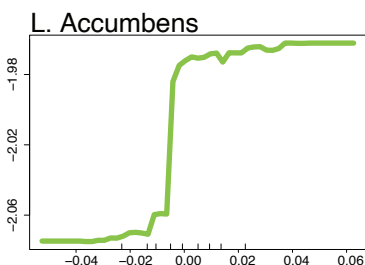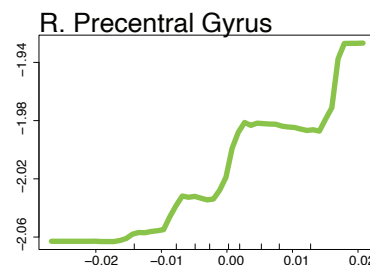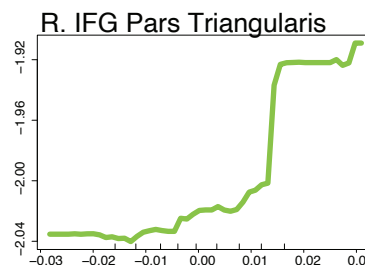

Regional Percent Change

Symptom Improvement

Supplement: Supplementary file 6 — Supplementary Figure S5 Outline of model using only regional volumetric changes as predictors. Top: Locations of top three important brain regional change predictors illustrated in Montreal Neurological Institute (MNI) space. Bottom: Partial dependence plots illustrating the expected degree of symptom dimension change (y‐axis) for observed values of the important regional volumetric change predictors (x‐axis) while holding all other model predictors at their observed median values [file HBM-42-5322-s002.pdf]
