# Supplementary material for: Accounting for symptom heterogeneity can improve neuroimaging models of antidepressant response after electroconvulsive therapy
Source: Hum Brain Mapp. 2021 Aug 13;42(16):5322–33. doi: 10.1002/hbm.25620 (PMC8519875; doi:10.1002/hbm.25620)
Supplement: Supplementary file 7 — Supplementary Table S1 Bilateral regions included as predictors [file HBM-42-5322-s005.docx]

| **Supplementary Table 1. Bilateral regions included as predictors** | |
| --- | --- |
| **Cortical** | **Subcortical** |
| superior temporal sulcus | accumbens |
| caudal anterior cingulate | amygdala |
| caudal middle frontal | caudate |
| cuneus | hippocampus (whole) |
| entorhinal | hippocampus body |
| frontal pole | hippocampus head |
| fusiform | hippocampus tail |
| inferior parietal | inferior lateral ventricle |
| inferior temporal | lateral ventricle |
| isthmus cingulate | pallidum |
| lateral occipital | putamen |
| lateral orbitofrontal | thalamus |
| lingual |  |
| medial orbitofrontal |  |
| middle temporal |  |
| paracentral |  |
| parahippocampal |  |
| pars opercularis |  |
| pars orbitalis |  |
| pars triangularis |  |
| pericalcarine |  |
| postcentral |  |
| posterior cingulate |  |
| precentral |  |
| precuneus |  |
| rostral anterior cingulate |  |
| rostral middle frontal |  |
| superior frontal |  |
| superior parietal |  |
| superior temporal |  |
| supramarginal |  |
| temporal pole |  |
| transverse temporal |  |
